# The pharmacology underlying the unique antipsychotic efficacy of clozapine

**DOI:** 10.1177/02698811251365177

**Published:** 2025-08-19

**Authors:** Gavin P Reynolds

**Affiliations:** 1 Biomolecular Sciences Research Centre, Sheffield Hallam University, Sheffield, UK; 2 Rotherham, Doncaster and South Humber NHS Foundation Trust, Doncaster, UK

**Keywords:** Clozapine, treatment resistance, hypersalivation, alpha2 adrenoreceptors, antipsychotic

## Abstract

Clozapine is unique in being the only recourse for people with schizophrenia not responding to conventional pharmacotherapy with dopamine D2 antagonists and partial agonists. Yet, after half a century of use, the underlying mechanism of clozapine’s relatively greater efficacy remains elusive. There have been many hypotheses relating to various neurotransmitter receptors that have not withstood further study, and some that have not been fully investigated. The recent introduction of the xanomeline-trospium combination for the treatment of schizophrenia has renewed interest in muscarinic receptor mechanisms; like xanomeline, clozapine and particularly its metabolite norclozapine reportedly have partial agonist actions at some muscarinic receptor subtypes. In their recent article, Morrison et al. draw attention to this by highlighting hypersalivation, a common feature of clozapine treatment that is not shared by other antipsychotic agents which they suggest to be a result of muscarinic receptor agonism. However the relatively weak muscarinic activity of clozapine, low brain availability of norclozapine and clinical findings from xanomeline combine to provide little support for muscarinic mechanisms underlying the greater efficacy of clozapine. An alternative hypothesis is that of alpha2 adrenergic receptor antagonism, a feature of clozapine pharmacology that may also contribute to clozapine-induced hypersalivation. Clinical findings with adjunctive alpha2 antagonists demonstrate clozapine-like improvements in antipsychotic efficacy, while both preclinical studies with specific alpha2C antagonists and the relatively high and selective antagonism of alpha2C receptors by clozapine provide support for this mechanism for clozapine’s unique efficacy.

## Introduction

Current management of schizophrenia by pharmacotherapy is limited. Only a minority of people with schizophrenia respond optimally to the available antipsychotic drugs that act as antagonists or partial agonists at the dopamine D2 receptor. A similar proportion respond so poorly that they are described as having treatment-resistant schizophrenia. Fortunately for some of these people, symptom relief can be obtained from treatment with clozapine. This drug has been considered unique in its efficacy in up to 50% of people in this group although, of those who do respond well, limiting side effects can sometimes prevent continued treatment. The severe and potentially fatal consequences of clozapine therapy include agranulocytosis, myocarditis and cardiomyopathy, and intestinal obstruction. Less acute but concerning adverse effects include weight gain, with consequent risk of cardiovascular disease and diabetes, and hypersalivation.

Despite its availability for over half a century, clozapine’s efficacy has not been replicated in more recently developed drugs. While the newer antipsychotic agents acting on dopamine systems are now generally better tolerated than the first-generation drugs, with a reduction in motor and hormonal side effects, equivalent improvements in the efficacy of symptom relief remain elusive.

It is important to recognise that the initial premise of a unique efficacy of clozapine in treatment resistance is not unequivocal. While a recent metaanalysis confirms clozapine’s superiority in patients having proved unresponsive to two prior trials of conventional antipsychotic drugs (i.e. the licensed indication for clozapine’s use), in a broader definition of treatment resistance olanzapine is shown to have similar efficacy (Dong et al., 2024). This likely relates to the understanding that treatment resistance in schizophrenia is not homogeneous and there are likely different patterns of treatment resistance (Lally et al., 2016) with differing pathophysiology. Nevertheless clozapine is unique in being the only drug with a licence for treatment of schizophrenia in patients unresponsive to, or intolerant of, conventional antipsychotic drugs. While there are other important clinical features of clozapine, notably its antisuicidal efficacy (Masdrakis and Baldwin, 2023), this article will focus on its pharmacology in the context of its licensed use in treatment resistance.

Clozapine has a complex pharmacology with actions at multiple neurotransmitter receptors. This is, in part, related to its relatively low affinity for the D2 receptor and consequent necessity for the relatively high doses used. Thus any receptor for which clozapine has an affinity at or above that for D2 is likely to be substantially occupied at normal clinical doses – most other antipsychotic agents typically show 10-fold (e.g. olanzapine) to 100-fold (e.g. risperidone) greater affinity at the D2 receptor, which will restrict drug action at other relatively low-affinity receptors (Zhou et al., 2022).

An understanding of the pharmacological basis of the particular efficacy of clozapine would be an invaluable step towards developing an equally effective antipsychotic agent without such limiting adverse effects. That understanding continues to elude us; several previous hypotheses have been based on selective effects at dopamine receptors, including at D2 splice variants (Malmberg et al., 1993), at the D4 subtype (van Tol et al., 1991) and at D1 receptors (Coward et al., 1989). These and other proposed mechanisms have been found either to be features of other antipsychotic agents or to be unsupported by the effects of drugs with greater receptor selectivity. However, in a recent Perspectives article in this journal, Morrison et al. (2025) have attempted to identify this unique pharmacological mechanism of clozapine by drawing attention to another feature of clozapine’s action which differentiates it from other antipsychotic drugs, namely the high incidence of hypersalivation. They propose this to be a consequence of action at muscarinic receptors which may also underlie clozapine’s efficacy in relief of symptoms in people with otherwise treatment-resistant psychosis.

This is an interesting hypothesis worth exploring further, particularly in the light of the recent introduction of the xanomeline-trospium combination as a novel antipsychotic agent with a muscarinic partial agonist action in the brain.

## The muscarinic hypothesis

Clozapine, along with several other first- and second-generation antipsychotics, has a high affinity for muscarinic receptors. Muscarinic acetylcholine antagonism is classically associated with hyposalivation; therefore clozapine-induced hypersalivation has, with some experimental evidence, been considered a muscarinic partial agonist action. The M3 subtype is primarily involved in the control of salivary secretion, with additional involvement of M1 and M4 (Abrams et al., 2006); it is these latter two receptors which are most implicated in the muscarinic action of clozapine in the CNS. However, clozapine itself has at most only weak agonist effects at the muscarinic receptors; the mechanism is more likely related to the major metabolite N-desmethylclozapine (norclozapine) which shows partial agonism at several of the muscarinic receptor subtypes, consistently with a higher percent efficacy, if rather lower affinity, than exhibited by clozapine (Lameh et al., 2007). Plasma levels of this metabolite, rather than clozapine, reportedly correlate with severity of salivation (Ishikawa et al., 2020), suggesting its role in this adverse effect.

The idea that muscarinic activity may also contribute to the particular efficacy of clozapine in treatment resistance is not new (e.g. Davies et al., 2005). These authors proposed the unique muscarinic partial agonism of norclozapine to be a component of a multifactorial pharmacological mechanism underlying clozapine’s efficacy. However, whether the peripheral actions of norclozapine can be extrapolated to the CNS is highly questionable. While norclozapine concentrations may approach those of clozapine in the blood, the same is unlikely to be true in the brain which, in the rat, accumulates clozapine at over 5-fold greater concentrations (Weigmann et al., 1999). This led to the conclusion that the consequently lower brain concentration of norclozapine indicates it is unlikely to play a role in any centrally-mediated effects of clozapine treatment.

That there is an interaction between cholinergic and dopaminergic systems is well established; antimuscarinic agents have long been used to ameliorate the motor side effects of D2 antagonism. In relation to the antipsychotic effect of muscarinic drugs, activation of the M4 subtype has been most implicated (Paul et al., 2024). This site acts primarily as an inhibitory autoreceptor on cholinergic neurons, both those modulating striatal output and those projecting to midbrain dopaminergic neurons.

The very recent introduction of xanomeline formulated in combination with trospium, a peripheral muscarinic antagonist aiming to minimise cholinergic side effects, provides the opportunity to further explore the muscarinic hypothesis of clozapine’s action. Xanomeline has much in common with norclozapine; it also acts as an M1 and M4 partial agonist, albeit with somewhat greater intrinsic efficacy (Odagaki et al., 2016). However there is little to support it as offering more than current antipsychotic treatments other than a different side effect profile. In a recent editorial, Javitt (2025) has summarised xanomeline’s overall antipsychotic efficacy as having an effect size comparable to common second-generation drugs, and somewhat less than that of clozapine. No significant overall procognitive effect was observed, quantified at rather less than that seen with current antipsychotic treatments, although there is some evidence for an effect in more severely cognitively-impaired subjects. It seems very likely that the antipsychotic effect of xanomeline relies primarily on an antidopaminergic action mediated by striatal muscarinic receptors (Reynolds, 2025).

These findings provide no support for a muscarinic hypothesis of clozapine’s particular efficacy, although we cannot rule out a possible synergism with other pharmacological mechanisms. Nevertheless, there are other, arguably stronger, hypotheses, one of which is an action at alpha2 adrenergic receptors.

## The alpha2 adrenoreceptor hypothesis

Some 30 years ago, Nutt (1994) proposed antagonism at alpha2 adrenoceptors as an action underlying antipsychotic atypicality. This no longer appears a general mechanism of second-generation antipsychotic drugs which have subsequently increased in number with no more than minor differences in efficacy, most having no substantial affinity for these sites. However, clinical support for this argument came from Litman et al. (1993), who found that the alpha2 antagonist idazoxan could enhance antipsychotic treatment response. This was further investigated in a controlled trial of treatment-resistant patients, demonstrating that clinical response to the D2 antagonist fluphenazine could be increased by adjunctive idazoxan (Litman et al., 1996), which, in a subgroup of patients, compared favourably with the effects of clozapine, indicating a mechanism possibly contributing to clozapine’s unique efficacy. This conclusion has also been drawn from preclinical studies showing how idazoxan enhances the potential antipsychotic effects of risperidone (Marcus et al., 2010).

There is further clinical evidence supporting alpha2 receptor antagonism as a valuable feature of clozapine’s pharmacology. Mirtazapine is an antidepressant drug with high affinity for alpha2 adrenergic receptors, an effect considered to contribute to its antidepressant efficacy. Several controlled trials have assessed mirtazapine as an adjunct to antipsychotic treatment, with reports of substantial symptom improvements (e.g. Berk et al., 2001; Joffe et al., 2009), although not always consistently (Berk et al., 2009). While these effects of mirtazapine are mainly on negative symptoms and cognition (Stenberg et al., 2010), it is notable that mirtazapine has not been tested in quite the same circumstances as clozapine would be prescribed, that is, in patients not responding to two prior antipsychotic treatments.

Interestingly, alpha2 antagonism has also been implicated in clozapine-induced hypersalivation. While a peripheral action on muscarinic receptors may indeed be important in drug effects on salivation, it has been shown that alpha2 receptors in the CNS provide a tonic inhibition of muscarinic agonist-mediated salivation (Takakura et al., 2003). That this may be important in clozapine’s action is indicated by the ability of lofexidine, an alpha2A/2C agonist, to ameliorate clozapine-induced hypersalivation (Corrigan et al., 1995). Furthermore, a pharmacogenetic study of clozapine-induced sialorrhea identified an association with a functional alpha2A receptor gene polymorphism (rs1800544) but found no relationship with polymorphisms in muscarinic M1 and M3 receptor genes (Solismaa et al., 2014). Of course, while these lines of evidence indicate the involvement of alpha2 receptors in the control of clozapine-induced hypersalivation, they do not prove that this is clozapine’s mechanism of action. This mechanism, therefore, is not fully understood; a useful working hypothesis might be that there is a synergistic effect of central alpha2 antagonism in enhancing the consequences of the relatively weak agonist effects of clozapine/norclozapine at muscarinic receptors involved in salivary control.

Of the alpha2 adrenergic receptors, alpha2A and 2C are the main subtypes present in the human brain, with 2A accounting for most alpha2 receptors and found in the majority in the frontal cortex, while 2C is the main subtype in the striatum (Blake et al., 1998). In some behavioural aspects they appear to have opposing functions; for example, genetic knock-out of the 2A subtype reduces cognitive performance while knock-out of the 2C subtype can enhance cognition (reviewed in Fragola et al., 2023). This implies a potential role for alpha2C antagonism (Uys et al., 2017) or alpha2A agonism (Fragola et al., 2023) in enhancing neurobehavioural function.

We investigated the binding of several antipsychotics to alpha2 receptors in human brain tissue, identifying differential affinities at alpha2A and 2C subtypes; notably only clozapine demonstrated an affinity for the alpha2C receptor which was not only greater than that for the alpha2A site but also substantially higher than at the D2 receptor (Blake et al., 1998). Supporting a specific functional effect of clozapine at the alpha2C site, experimental studies showed that clozapine, but not risperidone or haloperidol, could upregulate Adra2c gene expression in the rat frontal cortex without effects on Adra2a (Brocos-Mosquera et al., 2021). Uys et al. (2017) have summarised further pharmacological findings from several animal models inducing schizophrenia-like behaviours, showing how selective antagonism at the alpha2C site can ameliorate behavioural deficits and enhance the effect of haloperidol equivalent to that of clozapine.

## Conclusion

From this brief assessment of two aspects of the pharmacology of clozapine, it appears unlikely that actions at muscarinic acetylcholine receptors underlie the clinical advantage in efficacy that this drug demonstrates. On the other hand, a mechanism involving alpha2 adrenoreceptors, particularly of the 2C subtype, provides perhaps the best hypothesis for clozapine’s unique action. The development of selective alpha2C receptor antagonists available for clinical investigation (e.g. Rinne et al., 2016) introduces the opportunity to determine whether adjunctive 2C antagonism might relieve symptoms in some patients resistant to conventional antipsychotic drug treatment. This could offer an alternative to clozapine while potentially avoiding many of its associated adverse effects.

There are important caveats to this conclusion. One is that we should not ignore the possibility that a synergistic action between two, or even more, receptor mechanisms is responsible for clozapine’s efficacy. Thus it would be unwise to rule out dopamine D2 antagonism as a necessary component of clozapine pharmacology; other actions, requiring a functional presynaptic serotonin system, may also be important (Yadav et al., 2011). Furthermore, while a full understanding of clozapine’s unique mechanism could theoretically lead to the development of a drug of equivalent efficacy with fewer limiting side effects, such a pharmacotherapy will still leave unrelieved symptoms in many people with a diagnosis of psychotic illness. Clozapine is effective in typically only one half of those people with schizophrenia who meet criteria for treatment resistance; a substantial proportion therefore remain without any clear options for effective pharmacotherapy. Only further effort drawing on an understanding of the underlying neuropathology of schizophrenia, and its pathogenic mechanisms resulting from environmental and genetic risk, is going to enable a rational approach to effective therapeutic management.
